# A Novel Design of an Automatic Lighting Control System for a Wireless Sensor Network with Increased Sensor Lifetime and Reduced Sensor Numbers

**DOI:** 10.3390/s110908933

**Published:** 2011-09-16

**Authors:** Reza Mohamaddoust, Abolfazl Toroghi Haghighat, Mohamad Javad Motahari Sharif, Niccolo Capanni

**Affiliations:** 1 Faculty of Electrical, Computer & IT Engineering, Qazvin Azad University, Daneshgah Street, Qazvin 1416-34185, Iran; E-Mail: at_haghighat@yahoo.com; 2 Faculty of Computer Engineering & IT, Sadjad Institute of Higher Education, Mashhad 91375-4664, Iran; E-Mail: javadmotahari@gmail.com; 3 Faculty of Design and Technology, Robert Gordon University, St. Andrew Street, Aberdeen, AB25 1HG, Scotland, UK; E-Mail: n.capanni@rgu.ac.uk

**Keywords:** lighting control system, wireless sensor networks, energy conservation

## Abstract

Wireless sensor networks (WSN) are currently being applied to energy conservation applications such as light control. We propose a design for such a system called a Lighting Automatic Control System (LACS). The LACS system contains a centralized or distributed architecture determined by application requirements and space usage. The system optimizes the calculations and communications for lighting intensity, incorporates user illumination requirements according to their activities and performs adjustments based on external lighting effects in external sensor and external sensor-less architectures. Methods are proposed for reducing the number of sensors required and increasing the lifetime of those used, for considerably reduced energy consumption. Additionally we suggest methods for improving uniformity of illuminance distribution on a workplane’s surface, which improves user satisfaction. Finally simulation results are presented to verify the effectiveness of our design.

## 1. Introduction

Wireless sensor networks have various applications that include habit monitoring [1], wildfire monitoring [2], and navigation [3,4]. In today’s life, due to expanding social activities, people require reliable lighting during all hours of the day and night. Due to the limitations and rising cost of electrical energy production, it is becoming increasingly important to direct greater efforts into optimizing electrical energy utilization. Recently, wireless sensor networks have been applied to energy conservation applications such as light control [5–10]. The logic of lighting control systems may include factors such as daylight intensity, which is measured by light-sensitive sensors [7]. In [6] the authors defined several user requirements and cost functions. Their goal was to adjust lights to minimize the total cost of energy supplied. However, the result was applied to entertainment and media production systems. In [10], a tradeoff between energy consumption and users’ satisfaction in light control was studied. The authors applied utility functions which considered users’ location and lighting preferences so that illumination could be adjusted as to maximize the total utilities. However, it did not consider the fact that people may require different illumination levels for different activities. In [5,7], light control using wireless sensors to reduce energy consumption in commercial buildings is introduced. In this, lighting devices are adjusted depending on ambient daylight intensity.

In [8,9] a lighting control system is proposed that considers both users’ preferences and energy conservation. This system assumes that the location of each user is known via a wireless sensor that is carried by each user and that also detects local light intensity. An additional assumption is that there is no obstacle between whole lighting devices and fixed sensors. In [9] their model is designed for “point-link” light sources, such as LEDs.

In [11] a User Interface (UI) that improves the usability of the networked lighting system is proposed. This does not support the changes of lighting sources (internal and external resources) and in general it did not offer an exhaustive system. In [12], a design for automatic room light detection and control is proposed where a Home Light Control Module (HLCM) is installed in every light fixture of a family home. This system is intended for a home or a small office. In this each room uses one sensor and the placement of the sensors does not fall within the area illuminated by the lights which they control.

Measurement of light intensity is a parameter used in the decision-making process for different systems. The accuracy of measuring light intensity is therefore a contributing factor towards the accuracy of the whole system and consequently the amount of energy consumed. In [13], if lighting changes by a magnitude of 50 lx, the decision process performs an adjustment of (α = ± 50 lx), where (α) is the current system light intensity state. In all lighting systems which use them, the activity of permanently active sensors both increases energy consumption and reduces the lifetime of the sensors. The reduced sensor lifetime consequently increases the likelihood of faults in the system. Therefore energy management sensors are important in lighting control systems. However, energy management sensors are not discussed in many proposed systems such as [5,6,12].

In this paper we propose a Lighting Control System (LCS) based on wireless sensor networks. This system is called a Lighting Automatic Control System (LACS). The features of the LACS system are as follows:

- An increased sensor lifetime, with considerably reduced energy consumption.
- Recording of usage logs for multi-format report generation.
- Venue adaptability, e.g., it is applicable to various types of venues such as private and public residences, offices, lecture/conference halls, workshops, laboratories, libraries, retail premises, *etc*.
- Support of, and for, multiple simultaneous users.
- Minimization of communication and computing resources to moderate light intensity.
- Compatible with changes in external light source levels (sunlight and other environmental changes, e.g., street lighting, that have an effect on the room light levels).
- Magnitude of light intensity variations (α) is adopted as a parameter for changes in the current system state, e.g., adjustment of (α = ± a).

This paper is organized as follows. The first five sections present the LACS model and features. Sections 6 and 7 explain the concept of power management of sensors and reducing the number of sensors. Section 8 shows system configuration and initialization settings. Sections 9 and 10 contain discussion and simulation results. Section 11 draws the conclusions.

## 2. LACS System Architecture

**Figure 1.**
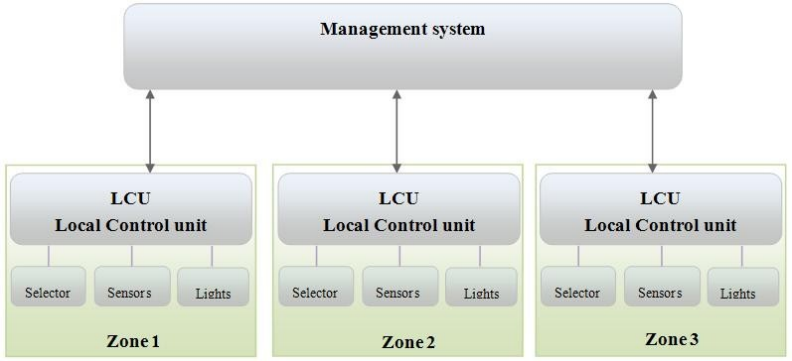
Lighting Automatic Control System (LACS) architecture.

The LACS shown in Figure 1 is composed of the management system and zones of operation. Each zone comprises a Local Control Unit (LCU) with its own activity selector, sensors and lighting units (Illumination Field). Each sensor monitors the illumination over a particular area, referred to as a “workplane”, which may have one or more lighting units. The individual components of the LACS system are as follows:

- Management system: the management software that is installed on a PC (or implemented as a standalone device). The management system controls the components of the system, records usage logs and constructs the system reports.
- LCU: each LCU module co-ordinates the information between the activity selector, sensors, and lighting units. Receives instructions from and reports to the management system. Additionally the LCU hosts an instance of the decision process for setting local light intensity.
- Activity selector: this component is the physical user interface by which users may communicate with the LACS by selecting their requirements.
- Sensors: each workplane has one sensor to measure light intensity. If light intensity changes across a programmable threshold tolerance value (± α), the sensor signals the LCU.
- Illumination Field: this is the set of lights that illuminate each workplane. The ballast on the lights is a dimmer.

In [14,15] a Wireless Sensor Network (WSN) management system is discussed. WSN management systems may be organized in centralized or distributed architectures. In a centralized management network, there is a single manager that collects information from all agents and controls the entire network. In the examples shown in this paper an LCU is an agent. A distributed management network has several managers, each responsible for a subnetwork and each communicates with the other managers. The specific management system is chosen based on the application running on the WSN.

**Figure 2.**
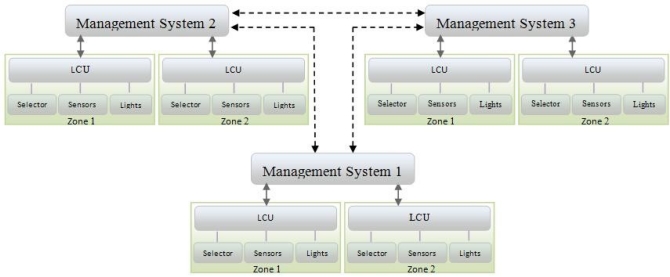
Distributed Lighting Automatic Control Systems.

A LACS system can contain various architectures based on application and space used. In Figure 1 an example of a centralized architecture is shown. The management system contains three zones, each of which has its own LCU and a number of workplanes, each workplane including a sensor and associated Illumination Field. Figure 2 shows a distributed LACS architecture. Each manager is independently responsible for several zones. In this architecture managers communicate with each other, for example to announce the change of decision policies or share statistical information.

In each LACS architecture the following assumptions are considered:

- Each workplane includes a sensor and its own activities.
- Only one user-selected activity can be enabled in each workplane at any time.
- The maximum required illuminance is known for all activities and set in the initial phase of system design.
- The maximum amount of required light for each workplane activity may be provided by the local Illumination Field, independent of possible non-system lighting.

## 3. System Performance

The operation of the LACS begins when a user chooses an activity from the Activity Selector. The current illuminance on the relevant workplane is sent to the LCU by the workplane’s sensor. Then the LCU compares the reported illuminance with the required illuminance of the selected activity. If there is insufficient light with respect to the normal activity value (α), a decision process is run in the LCU (described in Section 5). Otherwise the workplane’s light intensity is deemed suitable for the selected activity. The output of the decision process is the dimmer level that is sent by the LCU to light ballasts. By setting the amount of field light dims, a suitable light intensity is provided for the selected activities.

**Figure 3.**
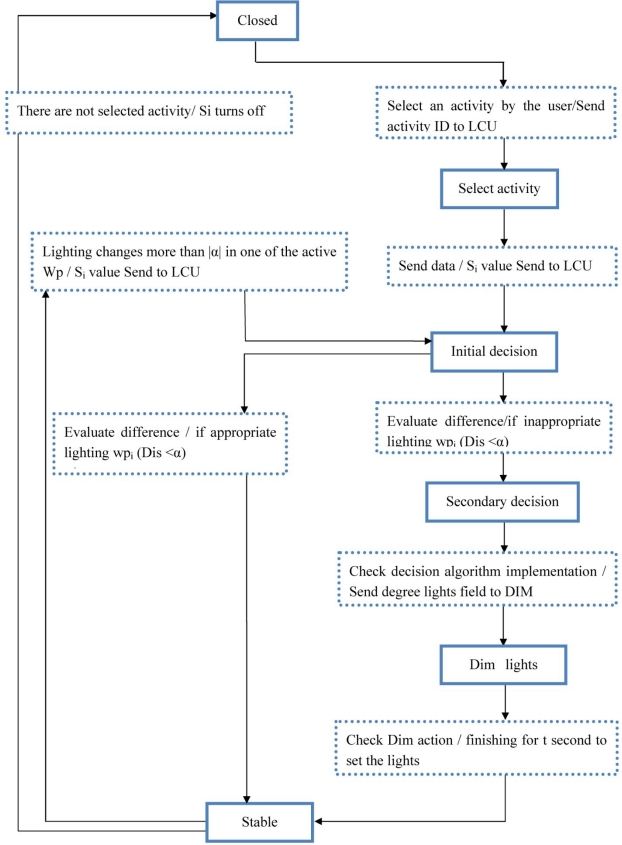
Lighting control finite state machine.

Figure 3 shows lighting control process modeling in the LACS. The necessary steps to control the lighting are shown in the form of a finite state machine. The six different states are listed in Table 1. In each “state” is a valid occurrence of some “events”, when a valid event occurs it results in a corresponding action (event/action).

If all of the workplanes are unused then the system is in its closed state. Whenever a user selects an activity, the system undergoes a transition to the selected activity state.

With the sending amount of the sensor (illuminance) for the activated workplane, the current amount of illuminance is compared with the illuminance value for the selected activity in the LCU (initial decision state). If the difference is more than α (|Dis| > α), the system goes to a secondary state for execution of the decision process. Dimmer levels of field lights are determined after the decision process. LCU regulates the intensity of field lights by sending dimmer levels to the light ballasts. Then, the system is placed in the stable state.

When system is in the stable state, each active workplane’s sensors measure the level of workplane lighting on a periodic basis. If the light intensity varies by ±α, due to external light sources, the system transitions from the stable state to the initial decision state. As can be seen in Figure 3, a system with inactivity in all its workplanes undergoes a transition from the stable to closed state.

## 4. Lighting Calculations for Light Intensity Prediction

With an optical sensor, or through lighting calculations formulas, point illuminance can be obtained. In this paper we present another method for predicting illuminance at a point. This is described in Section 5. The purpose of these calculations is to predict the level of workplane lighting in an internal environment.

**Figure 4.**
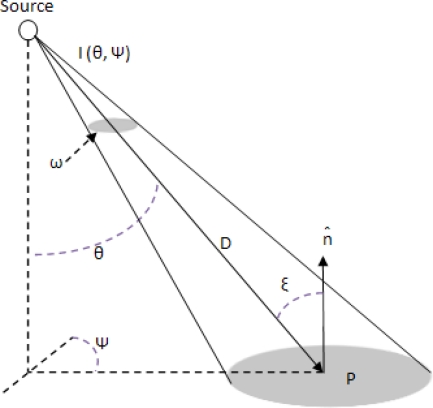
Light source in spherical coordinates.

The illuminance *E* produced on an area *A* centered at a point *P* is related to the luminous intensity of a light source, *I*(*θ*,*ψ*), as follows. Given the intensity distribution of the light source in spherical coordinates (*θ*,*ψ*), the geometric arrangement is shown in Figure 4. Equation (1) is the fundamental equation of flux transfer, the inverse square cosine law [16]:

(1) $$E=\frac{I(\theta ,\psi )\;\text{cos}\;\xi }{D^{2}}$$

where *D* = distance between the source and point *P*; *ξ* = angle between the normal (*n̂*) to the point *P* and direction of the distance *D; E* obtained in Equation (1) indicates the illuminance at a certain measurement point by a certain light. When there are a number of lights, the illuminance of a certain measured point is expressed by the summation of the illuminance given by each light [11]. The illuminance *E* depends on the illumination angle, *D* and other parameters in Equation (1) which are the constants that are uniquely set when the light and measurement point to simulate are determined.

As shown in Figure 4, the illuminance of at any point can be obtained through a light sensor or by the lighting calculation formulas. The effect of lighting intensity can be calculated for each point and each light source. By this method the effect of lighting from external and internal sources may be differentiated at any point. If light intensity at one point is provided only from the lights and external lighting sources do not have an affect, this method can predict a point illuminance. In general, the desired point and place of the lights are assumed fixed, so illumination angles and distance to the light sources are fixed, and the only variable factor is the dimmer level of each light source. Consequently, we can obtain the relation between the dimmer levels and the lighting intensity at one point without optical sensors, and even without using complex lighting calculation formulas. This method is described completely in Section 5.

## 5. LACS Decision Process

**Figure 5.**
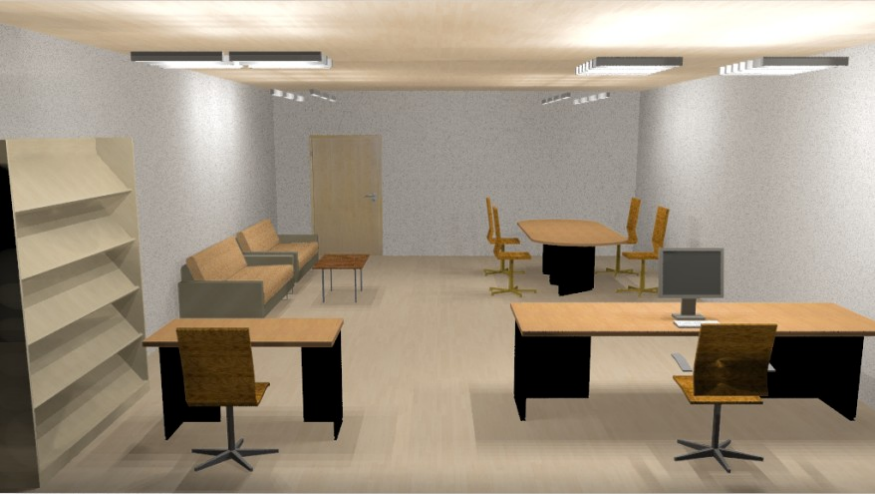
Workplane overlap from Illumination Fields.

When a zone contains more than one workplane, lighting from Illumination Fields may overlap and influence other workplanes. Figure 5 shows a sample of workplanes with their different lighting effects. All activities are carried out in four workplanes. These are: meeting desk (WP1, top right), waiting section (WP2, top left), journal study area (WP3, bottom left) and the secretary’s desk (WP4, bottom right).

Consider the scenario that all workplanes are initially inactive and that external light sources have no effect on the system lighting. After a while an activity is selected that relates to WP1 and requires 300 lx. When the lights of WP1 are turned on, WP2’s sensor detects 76 lx. If at WP2 an activity with a requirement of 200 lx is selected, then WP2’s lights should only provide 124 lx (not 200 lx) due to the 76 lx spillover from WP1’s lights. However by increasing the light output to 124 lx from WP2’s light, a spill over to WP1 of 34 lx occurs. Now WP1’s sensor detects 334 lx, while for the current activity 300 lx is sufficient. This causes WP1 to reduce its output by 34 lx and therefore the spill over to WP2 is reduced by 8 lx. This process continues until the required levels are reached for both workplanes. When all active workplanes have differences in their own requirements vs. supply levels (±α), the system continue to adjust until it reaches the stable state. We term this feedback process “sequential lighting changes”.

**Figure 6.**
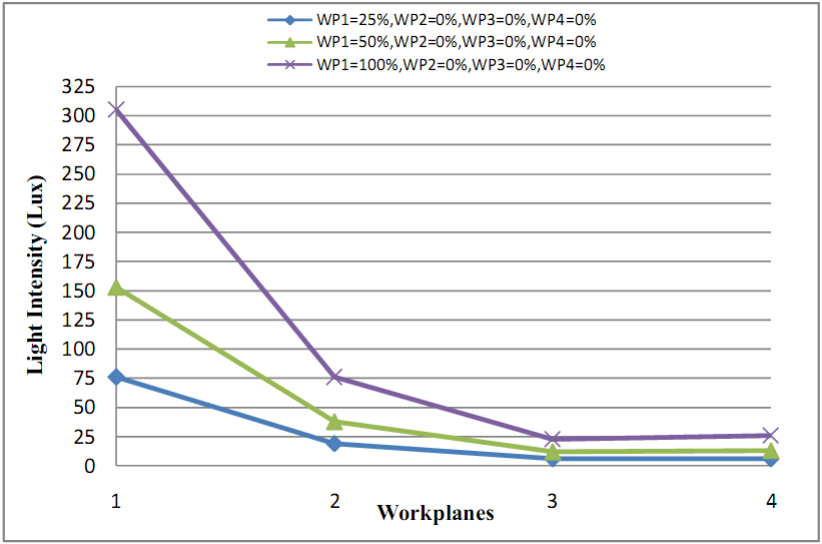
Lighting overspill from workplanes.

We suggest a method to solve the problem of “sequential lighting changes”. This is based on a decision process. First we define some concepts that are used in this solution. An illumination lighting effect dependency table is constructed by calculating the calculated levels of lighting on each workplane. For example, Table 2 lists the levels required for the four workplane scenario shown in Figure 5. Each row is related to a workplane and each column to an Illumination Field. The first entry in the first column is for the case that Illumination Field 1 has dimmer levels set to 100% and the other illumination fields set to 0%. Other rows of this column are related to spill over from Illumination Field 1’s lighting on the other workplanes. The effect of each Illumination Field on the other workplanes is shown in the remaining columns. In Figure 6 WP1’s Illumination Field is shown for dimmer levels at 25%, 50% and 100% while other workplanes are inactive (dimmer levels are 0%). In this figure the light intensity of all of workplanes in each state level is double that of the previous state level, so by knowing the effect of dimmer levels in an active workplane (while other workplanes are inactive) we can predict the amount of lighting effect that spills over to other workplanes with just a simple proportion at calculation. As can also be seen in Figure 6, with quadrupled lighting intensity values at 25%, a 100% dim state can be gained (without additional measurements).

As an acceptable limitation, the level of tolerance for activity lighting intensity in a specific workplane is given as α. For example, if the activity required 300 lx and α = ± 10 then the acceptable lighting intensity is 290–310 lx.

**Figure 7.**
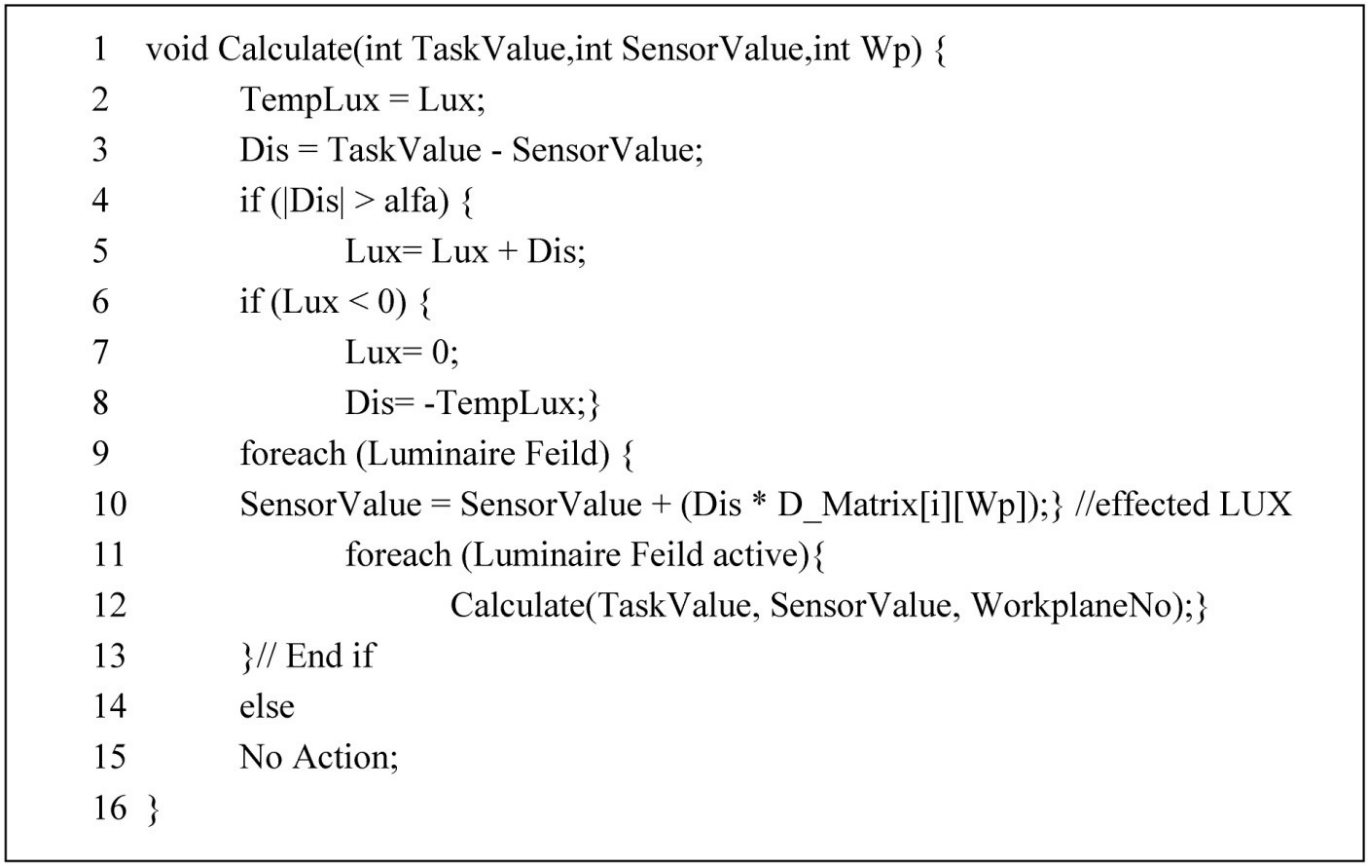
Pseudo code of Lighting Decision algorithm.

The above concepts have led to a decision process for which the pseudo code is shown in Figure 7. This method receives amounts of selected activity’s lighting intensity, current amount and workplanes number as three parameters. In line 2 the amount of current lighting in lux is saved in an auxiliary variable. The difference between the amount of current lighting and the expected value is stored in the (dis) variable. This value represents the degree of dimming. If this value exceeds α, then there is a requirement to adjust the lighting level. For changing it is sufficient to add the value of current lighting with the (dis) value. If the current lighting is more than expected lighting because (dis) is negative then lux is decreased in the summation operation, otherwise it is added to the current lighting. In lines 6 to 8 the amount of lighting is reduced to the maximum size of existing lighting. In lines 9 and 10 the dimming effect on other workplanes is calculated.

Matrix amounts D_Matrix[i][Workplane] are obtained from division of the dependency table value on Dependable_matrix[Workplane][Workplane]. In lines 11 and 12 the changes made on other workplanes are investigated so any pervious workplane adjustments do not change the amount of α. This form is implemented as a recursive procedure. On the other hand, because the number of workplanes in one zone is low (less than 32) implementation of the recursive algorithm does not cause much overhead. One important feature of this process is that it works with only one link established with the sensors.

## 6. Power Management of Sensors and Reducing the Number of Sensors

As a general approach skies may be divided into three categories: clear, partly cloudy, and overcast. When the sky is not completely overcast, the sky luminance distribution may change rapidly and by a large amount as the sun is alternately obscured, partly obscured, or fully revealed [16]. Consequently, if the external light sources have an effect on a room’s lighting, we cannot predict the illuminance of workplanes exactly because of the changing sunlight and weather conditions during the day.

In many places, due to geographical location, or due to the specific architecture of a building, external sources of lighting have little effect on interior lighting. After sunset external lighting is probably greatly diminished, except in rare cases where artificial night lighting has an effect.

Lots of lighting control systems, such [6,8,12], do not observe a difference between these two situation (external sources having an effect or not). In such situations, we propose methods for reducing the number of sensors (Sections 6.1 and 6.3) and sensor power management (Section 6.2). Generally these scenarios result in a reduction of the number of system sensors or a widespread increased sensor lifetime.

### 6.1. First Scenario

This method is used for the places where external light sources do not affect the interior space lighting; e.g., where there are thick curtains or no windows. In this situation we can predict illuminance on each workplane (WP), without using sensors. As mentioned, light intensity of each Illumination Field on each workplane is calculated by a lighting effect dependency table (Section 5). Consequently, the total effects of all illumination fields on each WP determine the illuminance of that WP. Therefore we can predict lighting intensity of each WP by pre-calculated dependency table without redoing complex calculations.

In [4,5,17] the number of workplanes or the number of users determines the number of required optical sensors. Thus, the overhead energy of the system increases. While with our method, we can predict the illuminance of some workplanes without the need for a sensor.

External lighting sources have no effect on interior lighting in Figure 5. In this room there are four WPs. By using the dependency table and illumination fields the required degree field lights adjustment can be obtained for a specific lighting intensity of each WP. So without using any sensors we can calculate lighting intensity of each WP in the LCU. After initialization phase (Section 9) can start reducing the number of sensors.

### 6.2. Second Scenario

If the Sun is the only external light source, then the LCU can use the known sunrise and sunset times to turn off sensors at sunset, and turn them on at sunrise (or at the first selected activity of the day). After sunset external sources have no effect and the system can therefore control the interior lighting without the need of sensors. In [6,8,12,17] sensors are on at all hours. Sunrise and sunset times based on geographical position and date can implemented in a LCU or system management.

### 6.3. Third Scenario

Daylight entering a building through apertures in the external fabric; such as windows, roof lights, light tunnels, *etc*. contributes to a Daylight Factor (DF). This is the ratio of the illuminance at a point on an interior plane, generally the horizontal work plane to the illuminance on an exterior horizontal plane produced by an unobstructed hemisphere of the same sky. The DF is applicable only when the external luminance distribution is known or can be reasonably estimated:

(2) $$\mathrm{DF}=\frac{E_{I}}{E_{H}}\cdot 100\%$$

where *E_I_* is the indoor illuminance at a given point and *E_H_* is the unobstructed horizontal exterior luminance.

The daylight factor method is a low-precision procedure for determining the illuminance at any point in an interior space produced by a sky with a known luminance distribution. Direct sunlight is excluded. The method is generally used with uniform or Commission International de L’Eclairage (CIE) overcast skies [16]. CIE standard lists a set of luminance distributions, which model the sky under a wide range of conditions, from the heavily overcast sky to cloudless weather. It is intended for two purposes:

- To be a universal basis for the classification of measured sky luminance distributions.
- To give a method for calculating sky luminance in daylighting design procedures.

This Standard incorporates both the CIE Standard Clear Sky and the CIE Standard Overcast Sky, which are treated as particular cases of the General Sky. The Overcast Sky is retained as a separate formula because there are many calculation procedures that embody the mathematical formulation of this particular distribution [18].

Due to limitations and the low accuracy of DF, this method is not useful for LCU use. Instead of using the DF method, we used another easy method that starts at the initializing phase (Section 8) for categorizing workplanes.

**Figure 8.**
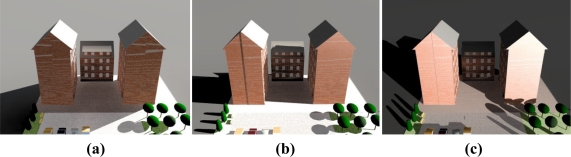
Large buildings shield a small building from sun light **(a)** 9 AM; **(b)** 12 AM; **(c)** 3 PM.

In the method used, initially all of the lights are off (or without change). After that the external lighting intensity is measured during the time that sky is clear. Simultaneously we examined and recorded the amount of WPs’ lighting intensity. After a day, the maximum amount of each WP is compared with threshold (*β*) and if the maximum is less than the threshold, then the external lighting is deemed to have little effect on that WP, where “*β*” is related to user satisfaction. These measurements can be done by software simulation, (see [19]). Samples of conditions of the places that include WPs with little effect from external lighting (and do not need sensors) are; places where their window have low degree of transmission, locations where the WPs are far from windows or when large obstacles (such as other buildings) shield the windows from external sources, as in Figure 8.

## 7. Setting Lighting Intensity on Workplanes Based on Multipoint Indicator

Each workplane (WP) includes one sensor (one of the assumption in the LACS system that is mentioned in Section 2). Each sensor functions as the illuminance indicator for its own WP. Then, the location of the sensor is the illuminance indicator point for its WP, and the decision algorithm uses this point as the illuminance for the WP surface.

For distributing illuminance on a WP’s surface that is more uniform, it is possible to use several different points as illuminance indicators of any WP. Therefore the decision algorithm uses these points as the illuminance of the WP’s surface. Thereafter, we suggested more methods for determining location of these points (WP’s illuminance indictors). These methods are used for workplanes that have sensors as well as ones than have not (as mentioned in Section 6).

### 7.1. Workplanes with Sensors

When external lighting does not have an effect on interior lighting, workplanes do not need to have sensors. In this condition, it must be determined which point of the workplanes should be selected as the illuminance indicator point.

To facilitate a decision on this, a lighting effect dependency table is constructed based on these points. As is the case for more uniform lighting distributing on a WP’s surface, we can use several locations as illuminance indicator points. Illuminance of one WP (*E_i_*) is obtained as an illuminance average weighting of these points and the dependency table is made from these values. This method caused more uniform lighting distribute and can achieve more satisfaction for the users. Equation (2) is a sample of multipoint average weighting on a WP. Impact factors have been considered to be equal (*λ_1_* = *λ_2_* = *λ_3_*). This method can also determine points for each WP where their lighting is more important for users by giving additional factors. Thus, the LACS system (decision algorithm) sets lighting intensity based on importance of indicator points:

(3) $$E_{i}=\lambda_{1}E_{p1}+\lambda_{2}Ep_{2}+\lambda_{3}E_{p3}\;\text{and}\;\lambda_{1}+\lambda_{2}+\lambda_{3}=1$$

Table 3(a–d) shows three dependency tables for three indicator points on each WP. Table 3(d) is calculated by combining elements of these three dependency tables considering Equation (1). The decision algorithm uses Table 3(d) as a main dependency table. In other words, in this method by average weighting elements of *K* (number of indicator points in each WP) a dependency table is mapped to a main dependency table.

### 7.2. Workplanes without Sensors

For the condition that external sources are effective on WPs, each WP has one sensor. However for sensed WP’s illuminance the algorithm needs to use several sensors for each WP. Increasing the number of sensors is not cost effective.

The size of a WP’s surface is often not big, and the effect of external light sources on all points of a WP is the same. With these considerations, each WP can use one sensor and several indicator points (virtual sensor). The Lighting Effect of internal lighting sources (Illumination Fields) on one point is calculated by a dependency table (*E_internal_*). The sensor sensed the illuminance of one point on the WP (*E_sensor_*). So the effect of external lighting intensity (*E_external_*) on a WP is calculated easily by Equation (3). As a result, each WP has one sensor while using K indicator points to have a suitable lighting distributing on the WP’s surface. This method requires the construction of a main dependency table similar to the “without sensor” method. Important indicator point factors in Equation (2) are calculated in the initializing phase. If external lighting intensity on a WP is distributed uniformly then λ is equal, otherwise, sensors points have the most *λ* than other points.

(4) $$E_{\mathrm{external}}=E_{\mathrm{sensor}}-E_{\mathrm{internal}}$$

## 8. Initializing Phase for Implementing LACS

In the Initializing phase, one should do basic configuration and initializing setting. For implementing a LACS, a set of parameters are defined and initialized according to the locational features. Importance parameters of this phase are as followed:

- Select system architecture based on the desired location.
- Measurement taken and geographical parameter extracted.
- Identify the locations corresponding to each workplane.
- Define the user activities and their required lighting intensity.
- Choose type of lights for internal light sources and simulate the lighting distribution.
- Categorize the workplanes.
- Evaluate and record the information for external sources, (e.g., sunset and sunrise time)
- Identify sensor placements and indicator points, set their factor.
- Construct affiliation table/s.

Other factors that are characterized as part of the initializing phase are determination of activities α and default value of lighting intensity, *etc*.

## 9. Discussion

A lot of parameters can be considered to affect the lifetime of an artificial light source. The following are two of the factors that can increase the lifetime of a lighting system:

1. Limiting the heat increase in a light.
2. Reducing the amount of switching (turning on and off) of a light.

In general higher temperatures will result in a decreased lamp lifetime. In the system that we have designed, the light intensity in a lamp is regulated, which results in a lower average temperature in a lamp than that of a lamp operating continuously at its highest capacity, so in systems where a lighting decrease causes a flow intensity decrease, the lifetime of the lamp will increase.

Another factor that causes the lifetime of a lamp to decrease is the number of times it is switched on or off. In general, the smaller this number the higher the lifetime of the lamp. The main reason for this effect is the repeated warming and cooling of the filament of the lamp. This is observed in all lamps that use a filament in their internal structure. For example in proposed system, when 97 percent of light can be fulfilled from external light sources, the lamps of a WP which would be used for obtaining 3 percent of light are not turned on. On the other hand, when a user leaves the working place for a short time, the lamps can go into a standby mode. In standby mode the lamp filaments work with their minimum flow and this will cause a decrease in number of switching for lamps. Alternatively for decreasing the number of switches a bigger α may be used. So updating and dimming in a system is reduced.

The best choice in implementing a LACS communication is the “ZigBee” protocol. The ZigBee alliance has developed a very low-cost & low-power consumption two-way wireless communications standard. Solutions adopting the ZigBee standard are embedded in consumer electronics, home and building automation, industrial controls, PC peripherals, medical sensor applications, toys, and games [20].

## 10. Simulation Results

**Figure 9.**
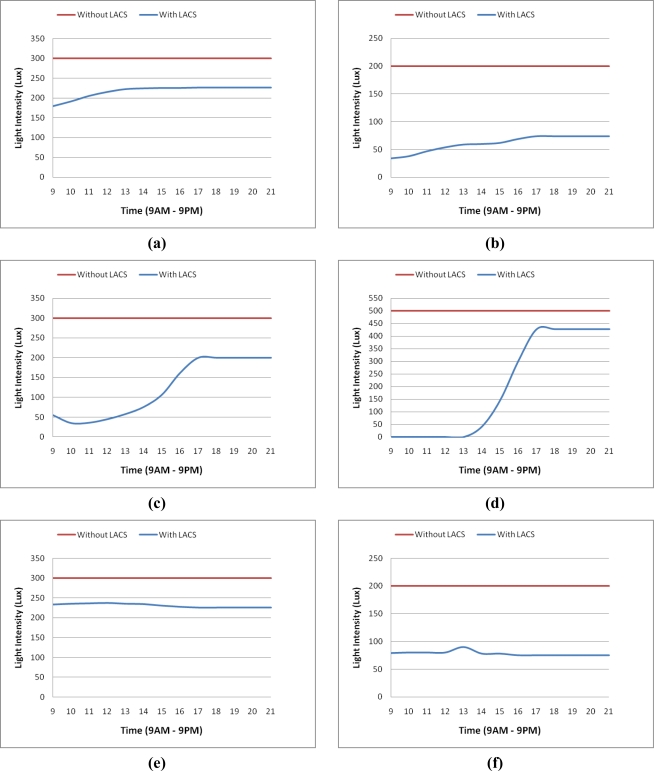

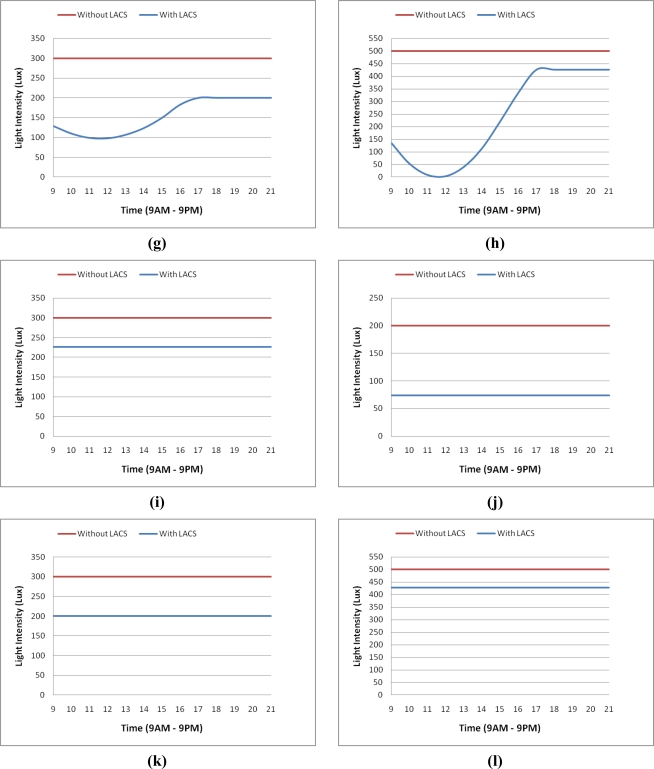
**(a)** WP1 and Clear sky; **(b)** WP2 and Clear sky; **(c)** WP3 and Clear sky; **(d)** WP4 and Clear sky; **(e)** WP1 and overcast sky; **(f)** WP2 and overcast sky; **(g)** WP3 and overcast sky; **(h)** WP4 and overcast sky; **(i)** WP1 and mixed sky; **(j)** WP2 and mixed sky; **(k)** WP3 and mixed sky; **(l)** WP4 and mixed sky.

For system simulations we have considered an office as shown in Figure 9. The dimensions of this are 5.48 m × 3.65 m × 2.74 m (L × W × H) and 34, 13 W OSRAM GmbH 72165 bulbs are used. This room is divided into four workplanes (discussed in Section 5).

**Figure 10.**
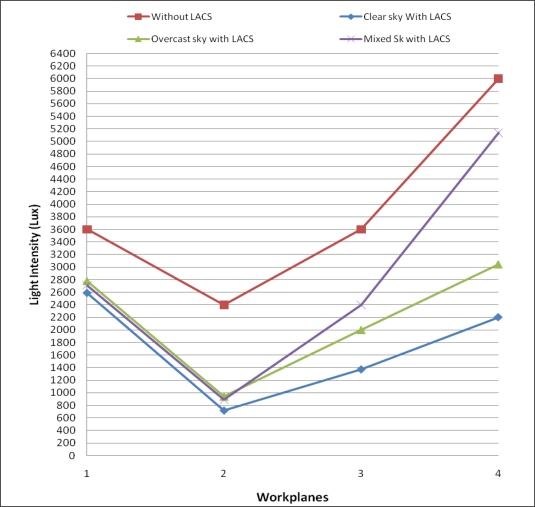
Total emitted light intensity on workplanes.

Consider a scenario that requires lighting intensity for conducted activities from 9:00 AM to 9:00 PM. WP1, WP2, WP3 and WP4 need 300 lx, 200 lx, 300 lx and 500 lx, respectively, for their activities. Figure 9 shows illuminance that is provided by the LACS on each wp at different hours and “clear sky”, “mixed sky” and “overcast” states. Total emitted light intensity on workplanes is shown in Figure 10. In the case that the current place did not use light from the LACS, the total of lighting intensity is the highest amount given.

Considered the second scenario that has a building with 20 floors and 40 units, and that all units have two independent rooms (total 80 rooms), and each room is similar to Figure 5. Activities conducted in these rooms are similar to the first scenario. In this building, workplanes of 20 rooms receive no (or little) effect from external lighting and 40 rooms are in receipt of external lighting. Two workplanes of the other 20 rooms are affected by external lighting but another two workplanes are not effected. The total amount of emitted light intensity on workplanes from 9:00 AM to 9:00 PM without using LACS is 1,248,000 lx. The value with using LACS is 646,240 lx in clear sky, 731,340 lx in overcast sky and 890,880 lx in mixed sky. As considered, the total amount of emitted light intensity on workplanes with LACS than compared to those without LACS it has been reduced from 29% to 48%. Also, the number of sensors required for all rooms is 200 instead of using 320 sensors. This is a 37.5% reduction by using the reduced number of sensors method (Section 6).

Table 4 compares the total the number of sensors, control equipment, accuracy, user satisfaction and system distributed architecture with other designs [9,12]. As a result, our design, which has more integrated functions, shows advantages over other current designs.

## 11. Conclusions

In this paper we have proposed the design of a Lighting Automatic Control System (LACS) based on wireless sensor networks. The architecture of our system can be centralized or distributed. Our decision algorithm makes use of a constructed lighting effect dependency table which contains the calculated levels of lighting on each workplane. A decision process is developed which minimizes the communication and computing resources required to moderate light intensity. When external lighting does not have an effect on interior lighting, our system can adjust the lighting intensity of rooms without using sensors. We also give methods which reduce the number of sensors, effect sensor power management and improve uniform distribution of illuminance on a workplan’s surface. Finally we show the LACS design has superior integrated functions and shows advantages over other current designs.

## Figures and Tables

**Table 1.** Lighting control states.

**State**	**Description**
Closed	No activity is selected or pending (all workplanes are disabled)
Stable	Workplane lighting intensity is appropriate for the selected activity and there is at least one active workplane
Select activity	One activity is selected in one workplane and system is not stable
Initial decision	The LCU examines difference between the selected activity lighting and the current workplane lighting (|α| < Dis)
Secondary decision	Decision process system is implemented
Light regulation	Lights’ fields are dimmed based on the decision process results

**Table 2.** Lighting effect dependency table for the share-space office as shown in Figure 5.

**Workplanes**	**Illumination Field (lx)**			
	***IF 1***	***IF 2***	***IF 3***	***IF 4***
WP1	305	55.9	19.2	47.3
WP2	75.8	202	32.8	56.2
WP3	22.9	20.4	307	88.8
WP4	25.5	25.7	67.1	503

**Table 3.** Lighting effect dependency tables for multipoint indicator.

**Workplanes**	**Illumination Field (lx)**			
	***IF 1***	***IF 2***	***IF 3***	***IF 4***
WP1	305	55.9	19.2	47.3
WP2	75.8	202	32.8	56.2
WP3	22.9	20.4	307	88.8
WP4	25.5	25.7	67.1	503
**(a)**				

**Workplanes**	**Illumination Field (lx)**			
	***IF 1***	***IF 2***	***IF 3***	***IF 4***
WP1	292	61	19	47
WP2	80	194	33	58
WP3	23	21	317	103
WP4	25	25	56	514
**(b)**				

**Workplanes**	**Illumination Field (lx)**			
	***IF 1***	***IF 2***	***IF 3***	***IF 4***
WP1	300	54	18	44
WP2	80	205	30	53
WP3	22	19	310	89
WP4	27	27	67	491
**(c)**				

**Workplanes**	**Illumination Field (lx)**			
	***IF 1***	***IF 2***	***IF 3***	***IF 4***
WP1	299	57	18.7	46.1
WP2	78.6	200.3	31.9	55.7
WP3	22.6	20.1	311.3	93.6
WP4	25.8	25.9	63.4	502.7
**(d)**				

**Table 4.** **C**omparison of system architecture with current and LACS designs.

	**Design 1**	**Design 2**	**LACS**
**Number of sensors**	Number of rooms	Number of Users	From 0 to Number of WPs
**Sensor reduction mechanism**	No	No	Yes
**Accuracy**	Very low	High and constant	High and adjustable
**Decision algorithm**	Simple	Complex	Simple-medium
**Users Satisfaction**	Low	Trying to raise	High
**System Architecture**	Centralized and limited	Centralized and distributed, but limited	Centralized and distributed widely
**System Management**	No	Simple Management	Full Control
